# AI in conjunctivitis research: assessing ChatGPT and DeepSeek for etiology, intervention, and citation integrity via hallucination rate analysis

**DOI:** 10.3389/frai.2025.1579375

**Published:** 2025-08-20

**Authors:** Muhammad Hasnain, Khursheed Aurangzeb, Musaed Alhussein, Imran Ghani, Muhammad Hamza Mahmood

**Affiliations:** ^1^Department of Computer Science, Lahore Leads University, Lahore, Pakistan; ^2^Department of Computer Engineering, College of Computer and Information Sciences, King Saud University, Riyadh, Saudi Arabia; ^3^Department of Computer and Information Sciences, Virginia Military Institute, Lexington, KY, United States

**Keywords:** ChatGPT, comprehensiveness, DeepSeek, eye infection, prompts

## Abstract

**Introduction:**

The advent of large language models and their applications have gained significant attention due to their strengths in natural language processing.

**Methods:**

In this study, ChatGPT and DeepSeek are utilized as AI models to assist in diagnosis based on the responses generated to clinical questions. Furthermore, ChatGPT, Claude, and DeepSeek are used to analyze images to assess their potential diagnostic capabilities, applying the various sensitivity analyses described. We employ prompt engineering techniques and evaluate their abilities to generate high quality responses. We propose several prompts and use them to answer important information on conjunctivitis.

**Results:**

Our findings show that DeepSeek excels in offering precise and comprehensive information on specific topics related to conjunctivitis. DeepSeek provides detailed explanations and in depth medical insights. In contrast, the ChatGPT model provides generalized public information on the infection, which makes it more suitable for broader and less technical discussions. In this study, DeepSeek achieved a better performance with a 7% hallucination rate compared to ChatGPT's 13%. Claude demonstrated perfect 100% accuracy in binary classification, significantly outperforming ChatGPT's 62.5% accuracy.

**Discussion:**

DeepSeek showed limited performance in understanding images dataset on conjunctivitis. This comparative analysis serves as an insightful reference for scholars and health professionals applying these models in varying medical contexts.

## 1 Introduction

Conjunctivitis, also known as “pink eye,” is an ocular condition that can affect individuals of all ages. It has adverse impacts on public and private economies and their productivity (Gunay et al., 2015). Various antibiotics were prescribed for bacterial conjunctivitis, which are now not frequently used due to bacterial resistance and safety concerns (Karpecki et al., 2010). Healthcare practitioners cannot discriminate between the viral and bacterial infections of conjunctivitis, resulting in over 80% of patients receiving antibiotics. Recent reports indicate that over 900,000 antibiotic prescriptions were dispensed in the Netherlands, incurring a cost of $10.9 million (Rietveld et al., 2004).

**Figure 1:**
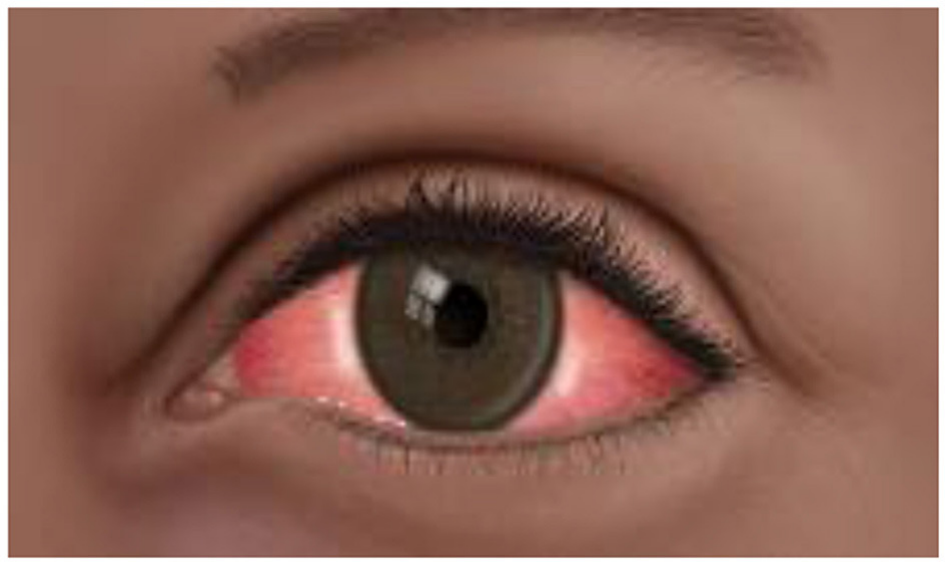
Pink eye conjunctivitis.

Recently, Pakistan faced a big health issue of conjunctivitis. We witnessed an alarming surge in infection in different areas of Pakistan. More than 86,133 cases were confirmed in Pakistan (Ahmed et al., 2024). This infection rapidly spread to other parts of the country. This issue demanded immediate attention from healthcare professionals, government officials, the research community, policymakers, and pharmaceuticals. Medical and research communities could play a big role in a coordinated and strategic way to overcome the prevalence and pressing issue of conjunctivitis infection (Alves et al., 2023). Figure 1 is the illustration of pink eye conjunctivitis.

Several factors contribute to the escalating cases of conjunctivitis. Notably, the lack of clean water and sanitation facilities can facilitate the spread of this highly contagious infection (Jasper et al., 2012). Moreover, insufficient education and public awareness regarding eye hygiene practices may contribute to its transmission. Recent research does not definitively establish conjunctivitis as the sole sign of a sexually transmitted infection (Abedifar et al., 2023). A recent study stated that the conjunctivitis rate was highest among children under 7 years of age. Viral conjunctivitis could be caused by contact (Hashmi et al., 2024). This study supports the observation that conjunctivitis is common in people of all ages. A child with no previous history of conjunctivitis could catch this infection from family members. Previous studies evidenced that bacterial conjunctivitis was more common in children than adults.

Large language models (LLMs) have several applications in clinical settings. LLMs show potential in image analysis when integrated with the computer-aided diagnosis (CAD). LLMs summarize information in natural language that help in diagnosis and generating health reports (Wang et al., 2024). In a recent research, researchers revealed that ChatGPT-4 and PaLM2 LLMs achieved better performance in answering questions on ocular surface diseases (Ling et al., 2025). However, ChatGPT-4 offered potential in improving the accuracy of question-answering and may serve as a valuable tool for medical professionals in future. Previous studies have not compared ChatGPT and emerging AI application such as DeepSeek on health topics. Most recent research employed variants of ChatGPT, such as ChatGPT 3.5 and ChatGPT 4.0, and compared their abilities to diagnose glaucoma in a ocular hypertension treatment study (OHTS) dataset (Raja et al., 2025). ChatGPT-4 achieved better accuracy in diagnosing glaucoma compared to ChatGPT 3.5. ChatGPT serves as a beneficial tool in exploring ocular hypertensive eye when specific information is available. However, none of the studies examined the role of ChatGPT-4 compared to DeepSeek, an emerging LLM, on conjunctivitis topics. Based on the available data on conjunctivitis, our study fills up this research gap by comparing the performance of ChatGPT 4.0 and DeepSeek on health topics related to conjunctivitis.

This study has the following contributions:

- This study aims to reveal how AI models may assist in recalling or identifying relevant factors that support the clinical diagnosis of conjunctivitis.
- It compares the performance of ChatGPT and DeepSeek on topics related to conjunctivitis infection and evaluates how the two models are powerful in giving precise and comprehensive information.
- We use the hallucination rate metric to test factual accuracies and compare the performance of ChatGPT and DeepSeek models.
- This study compares the capabilities of ChatGPT, Claude, and DeepSeek models in classifying healthy and conjunctivitis-affected eyes from a real-world dataset.

The layout of this study is as follows:

We detail research methods in Section 2. The results and their discussion are given in Section 3. The main points of this study are concluded in Section 4.

## 2 Literature review

Ophthalmology has experienced significant improvements from artificial intelligence (AI) in recent years through its enhanced management of eye infections. The Knowledge-Guided Diagnosis Model (KGDM) emerged from research (Chen et al., 2020) as an interpretable AI system to support infectious keratitis diagnosis. This model combines expert clinical understanding with data intelligence to generate visual reasoning systems that use AI-based biomarkers and comparable case searches. The KGDM exhibited excellent diagnostic capabilities across various datasets. At the same time, it improved medical worker performance by working alongside human experts, which presents significant potential for AI use in eye infection diagnosis.

The study by (Rajarajeshwari and Selvi 2024) conducted a detailed review of AI usage in major retinal conditions. The research emphasized early detection capabilities while assessing multiple diagnostic and structural anomaly detection algorithms for retinal diseases. According to the authors, future research should concentrate on boosting computational complexity while using existing public datasets to increase the reliability of AI-based detection methods.

The DATUM alpha study by (Odaibo et al. 2019) evaluated the cloud-based mobile AI technology for retinal disease detection within mobile health technology. The mobile application named Fluid Intelligence underwent testing to detect subretinal fluid and macular edema from optical coherence tomography (OCT) scans. The AI system demonstrated a weighted average sensitivity of 89.3% along with a specificity of 81.23%, indicating its value as a screening tool, particularly for underserved areas.

In a study (Yoo et al., 2019), authors analyzed current developments in central serous retinopathy (CSR) detection, which combines imaging with artificial intelligence methods. The researchers analyzed traditional imaging approaches such as OCT and fundus imaging alongside machine and deep learning methods. The study findings show that deep learning classifiers perform CSR detection with high accuracy while also achieving fast results and reliable outcomes. However, more research is necessary to optimize the computational performance and establish their effectiveness on open-source datasets.

ChatGPT supports graduates to score better than their peers from medical schools. ChatGPT and other similar applications provide interactive support, personalized learning, motivation boosting, and self-assessment (Gencer and Gencer, 2024). Consequently, most recent study presents a bibliographic analysis to show current research trends. The results show that ChatGPT, clinical management, natural language processing (NLP), Chatbots, and virtual reality indicate a shift toward addressing implications at a higher level for LLMs and their applications in healthcare (Gencer and Gencer, 2025). ChatGPT applications are widely discussed in the literature. One of the studies highlights applications for improving the effectiveness and efficiency of educational, clinical, and research work in medicine (Thirunavukarasu et al., 2023).

## 3 Research methods

### 3.1 ChatGPT 4.0

ChatGPT application adds to medical education clinical decision-making. A recent study examined the role of ChatGPT and its performance in assessing ophthalmology-related queries (Alexander et al., 2024). ChatGPT and its variants demonstrate a strong capability in handling medical knowledge. Even ChatGPT and Bard as large language models (LLMs) show remarkable performance in domains such as intelligence diagnostics (Caruccio et al., 2024a).

During times of crisis, we rely on invaluable information and support from generative artificial intelligence applications such as ChatGPT, which can promptly respond to inquiries posed to the application. In the context provided above, we posed several questions to ChatGPT for public awareness and education regarding conjunctivitis infection.

### 3.2 DeepSeek

DeepSeek was developed by a Chinese AI company, which can process users' queries at a low cost. Open source and cost-efficient DeepSeek is disrupting the conventional AI approaches and their applications in various fields (Krause, 2025). The basic architecture of DeepSeek is based on the transformer framework.

### 3.3 Claude

Anthropic released its AI model, Claude 3.5 Sonnet, on 21 June 2024. As per official statements, Claude 3.5 Sonnet is superior in performance compared to GPT-4, Gemini, and other predecessors. The Claude model provides rapid response to prompts for generating discharge summaries for kidney patients (Anthropic, 2024). It can handle complex medical tasks and effectively integrate with medical field (Jin et al., 2024). Research on the Claude model showed an improved performance using imaging data and medical history. It showed improved tumor, node, metastasis (TNM) classification results using the radiology reports of pancreatic cancer patients (Suzuki, 2025). Hence, we use the Claude model for classification task and compare its performance with ChatGPT and DeepSeek models.

### 3.4 Prompt engineering

The method of designing optimized input prompts functions such as prompt engineering to guide GPT models toward generating specific output content. The process of prompt engineering requires the creation of questions or instructions that match both model abilities and necessary task functions. Zero-shot prompt engineering enables the creation of prompts that allow the system to execute untrained functions without any supplemental fine-tuning operations. The generalization capabilities of the model allow this approach to work. When GPT receives text summarization or problem-solving requests before any specific training occurs, it operates through zero-shot prompting. The majority of studies used a zero-shot prompt engineering technique, followed by a few-shot technique. The few-shot technique outperforms a zero-shot technique in addressing multiple-choice questions, while its performance remains inconsistent in NLP tasks. A mixed prompt engineering technique has been reported to lead to a better performance than using any prompt engineering technique alone (Zaghir et al., 2024). Hence, we used a mix-up of zero-shot and few-shot prompt engineering techniques in this study. Prompt engineering may enhance the learning capability and generalization of an LLM on a small dataset and fully exploit the potential performance of a model.

### 3.5 Hallucination rate

The hallucination rate is used to quantify the proportion of Generative models' produced references, which are irrelevant, incorrect, and unsupportive by the available literature (Chelli et al., 2024). When LLMs are confronted with factual questions, hallucinations may occur, resulting in information that is factually incorrect but syntactically and grammatically correct. The hallucination rate is judged by using a systematic verification process based on PubMed and Google Scholar to ensure accuracy, legitimacy, and transparency. Each reference is verified by searching for its title in PubMed and Google Scholar databases (Aljamaan et al., 2024). In case of failing to reach a reference in the PubMed database, Google Scholar was the second choice for verification using a paper's title. Hallucination rate can be expressed as given in the Equation 1.


(1) $$\begin{array}{l} \text{Hallucination rate}=\frac{\text{No of fake or inaccurate references}}{\text{Total references generated}}\times 100 \end{array}$$

The hallucination rate is measured by conducting human evaluations. Annotators compare the model's generated output with ground truth references, thereby flagging fabricated, incorrect, or irrelevant instances of responses. Next, we calculate the hallucination rate by using the formula in Equation 1. We preferred using human evaluations over automated metrics, such as BLEU or ROUGE, because humans can detect contextual hallucination, which is overlooked by automated metrics.

### 3.6 Data synthesis

The process of data synthesis creates artificial datasets that replicate actual data to overcome data shortages and unbalanced distributions and protect sensitive information. The creation of realistic dataset succeeds through the application of algorithms alongside simulations and models, including Generative Adversarial Networks (GANs), and no synthetic dataset was employed in this study. Data synthesis enables important tasks such as model training and sensitive domain research while protecting actual data from disclosure.

### 3.7 Contour count

Eyelid contour abnormalities are widely reported in the literature. In a study, researchers reported that postoperative and preoperative digital images were used for eyelid analysis (Choudhary et al., 2016). A contour represents a curve that joins continuous points along a boundary with the same intensity or color. Thus, contour count is the number of distinct boundaries, which are detected in an eye image. The rationale behind using contour count as a feature of conjunctivitis relates to the iris, pupil, and sclera being smooth and distinct for a healthy eye. For conjunctivitis eye, inflammation or redness creates more edges or irregular shapes. When blood vessels become more prominent, it could increase the contour detected. Alongside contour count, the mean intensity feature is employed in this study to increase accuracy and reliability in results. Contour count and mean intensity features could help us differentiate between healthy eyes and those affected by conjunctivitis.

Computer vision directly interprets images, faces, and scenes and detects objects using convolutional neural networks (CNNs) and transformers. On the other hand, natural language processing (NLP) processes texts extracted from images using the optical character recognition (OCR) technique. NLP techniques are used to analyze words but not visuals. Computer vision understands pixels, while NLP relies on the pre-trained text. NLP allows computers to understand human languages such as Spanish and English, by converting letters into numbers using some algorithms (Manovich, 2021).

### 3.8 Mean intensity

Mean intensity as a feature to distinguish between healthy eye and eyes affected by conjunctivitis is used in this study. Mean intensity refers to the average pixel brightness within the eye region (Singh et al., 2021). A healthy eye provides moderate values of the mean intensity due to consistent illumination and reflectance. In conjunctivitis, vascular changes and inflammation alter reflectance, resulting in irregular or elevated intensity patterns. This feature is highly supportive for early detection and differentiation and provides the quantitative examination of tissue health.

### 3.9 Pre-processing and standardization

For LLMs used in this study, image classification involves converting visual information into textual prompts because of the text-based nature of these models. Pre-processing image data include resizing images and converting them to the textual description. Moreover, we standardize input information and ensure a uniform prompt structure. The input lines of a model are carefully constructed prompts that embed the clinical context and image-driven characteristics and allow a model to infer a reliable diagnostic classification from structured textual prompts.

### 3.10 Classification accuracy metric

#### 3.10.1 Accuracy

Accuracy is a measure that shows the proportion of accurate predictions relative to all predictions. The formula of accuracy measure is expressed in the Equation 2.


(2) $$\begin{array}{l} Accuracy=\frac{TP+TN\;}{TP+TN+FP+FN} \end{array}$$

#### 3.10.2 Precision

The precision measure is the ratio of correctly classified positive values to the estimated total number of positive samples measured. The precision is expressed in the following Equation 3.


(3) $$\begin{array}{l} Precision=\frac{TP}{TP+FP}\times \;100 \end{array}$$

#### 3.10.3 Recall

The recall measure is about determining the proportion of positive values, which are correctly classified. The recall formula is given in the Equation 4.


(4) $$\begin{array}{l} Recall=\frac{TP}{TP+FN}\times \;100 \end{array}$$

#### 3.10.4 F1-score

The F1-score can be described as a harmonic mean of precision and recall measures. Precision and recall equally contribute to the score and ensure the reliability of the classifier. This measure can be expressed in the following Equation 5.


(5) $$\begin{array}{l} F1-Score=2\;\left(\;\frac{Precison^{*}Recall}{Precision+Recall}\;\right) \end{array}$$

### 3.11 Ethical considerations

AI ethical concerns aim to establish three major standards related to design approaches and system usage, which consist of promoting fairness and transparency and maintaining accounting responsibilities. Three main ethical challenges stem from correcting data bias, protecting user privacy, and managing consent within sensitive application frameworks. Medical AI systems operating in eye infection management should first prioritize concentrating on patient protection combined with model transparency alongside equal technology access for all patients. The development of responsible innovation depends heavily on ethical guidelines together with regulatory compliance to build trust between stakeholders.

## 4 Results

### 4.1 Dataset description

This study employed a real-world Kaggle conjunctivitis dataset. The dataset contains 181 healthy eye's images and 177 infected images with conjunctivitis. This dataset can be accessed from the Kaggle website (Sofiya, 2025).

### 4.2 Results description

We asked targeted questions to evaluate the ChatGPT's capability in processing and analyzing diverse clinical information. Moreover, we assessed the ChatGPT's utility to manage the disease with multiple etiologies.

The first prompt was, “Is there any multifaceted approach to mitigate conjunctivitis infection?” In response to this prompt, ChatGPT's response was as follows:

A multifaceted approach can be employed to mitigate conjunctivitis. This includes ensuring access to clean water, promoting hygiene, providing education, enabling early diagnosis, establishing treatment protocols, implementing surveillance, administering vaccinations, fostering research collaboration, and advocating for policies. DeepSeek provides the following response for the same prompt. In addition to the response from DeepSeek, we compared the responses generated by DeepSeek to ChatGPT 4.0 in the following Table 1.

**Table 1:** Comparison of ChatGPT and DeepSeek on various aspects of conjunctivitis.

**Aspect**	**DeepSeek**	**ChatGPT 4.0**
Scope	Focused on conjunctivitis-specific prevention and management	Broader, addressing infectious diseases at a systemic level
Clean water	Not mentioned	Ensuring access to clean water for hygiene and sanitation
Hygiene	Emphasizes personal hygiene practices	Promotes hygiene at community and systemic levels
Education	Limited to public awareness about conjunctivitis	Includes broader education on infectious diseases and prevention
Early diagnosis	Focused on prompt medical consultation for conjunctivitis	Enabling early diagnosis for a wide range of infectious diseases
Treatment protocols	Specific to conjunctivitis medications	Establishes protocols for treating various infectious diseases
Surveillance	Not mentioned	Implements disease surveillance systems
Vaccinations	Not mentioned	Includes administering vaccinations for preventable diseases
Research collaboration	Not mentioned	Fosters collaboration for research on infectious diseases
Policy advocacy	Not mentioned	Advocates for policies to support public health initiatives

Based on the data in Table 1, we can make a difference between two AI models with the same prompt. DeepSeek provided us with specific information on conjunctivitis, while ChatGPT's reply was focused on a comprehensive framework for controlling infectious diseases. DeepSeek does not fully capture some systemic focus, such as access to clean water, surveillance, and policy advocacy. ChatGPT response incorporates vaccinations and research collaboration strategies, which are absent in a response from DeepSeek. Response from DeepSeek is primarily focused on individual and community-level actions to overcome the conjunctivitis infection.

**Figure 2:**
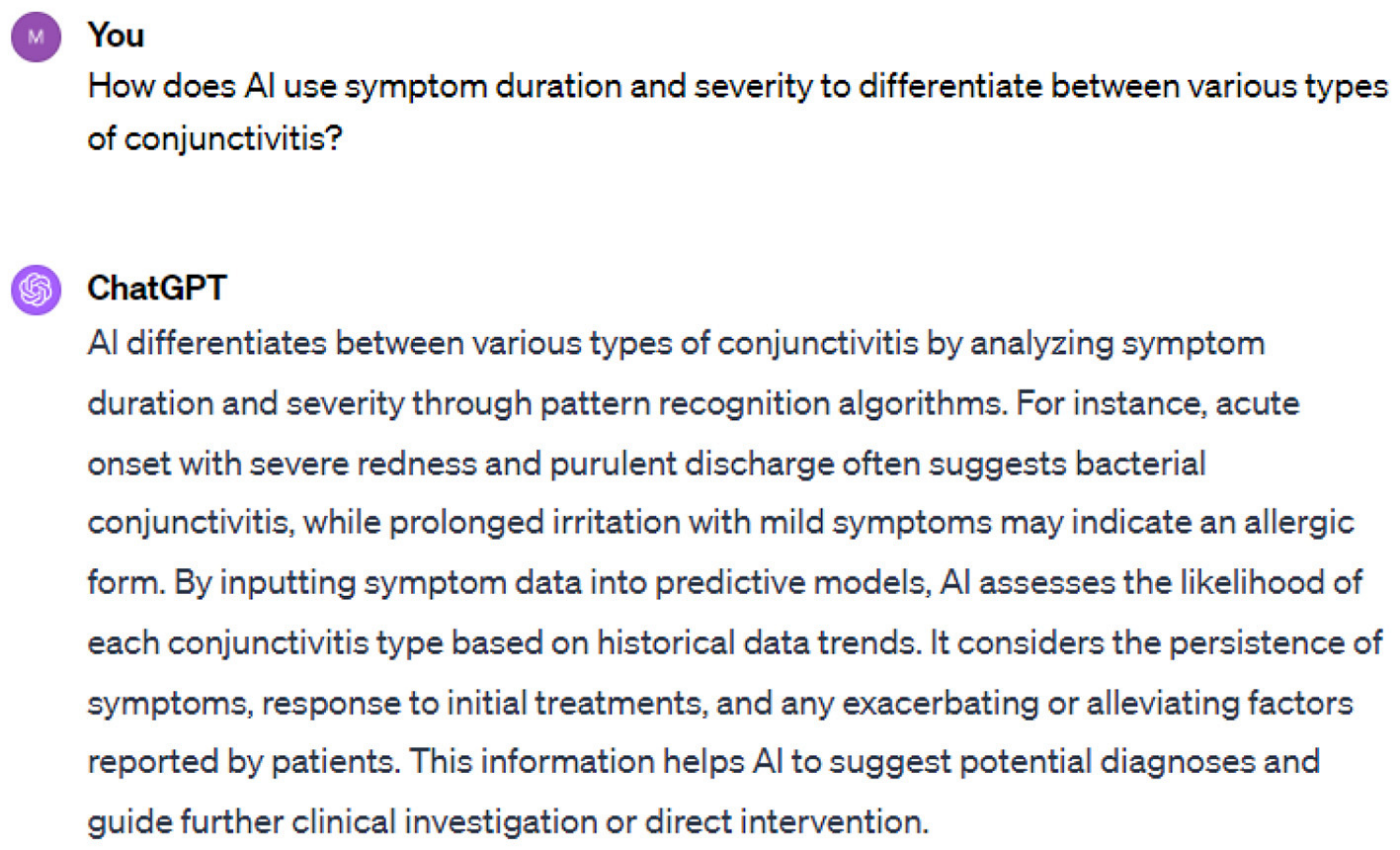
Prompt 2.

On the other hand, ChatGPT, in contrast to DeepSeek, provides broader systematic health measures that are common to other infections. Therefore, DeepSeek provides more precise information about health issues. Each measure given by DeepSeek is focused on either preventing or managing the health issue. DeepSeek recommends antihistamines for allergic conjunctivitis; however, it missed the mention of clean water in its response and instead responds with good hygiene, which depends on the clean water. DeepSeek analyzes thoroughly while answering the prompt and significantly checks the context of the prompt before responding to the users. Therefore, DeepSeek is superior to ChatGPT in terms of contextual values and provides precise, practical, and directly applicable information. For public health, ChatGPT is superior since it reveals information on addressing systematic gaps, scalability, and multi-disease resilience. Next, we have prompt 2 and its response from ChatGPT 4.0 in the Figure 2.

**Figure 3:**
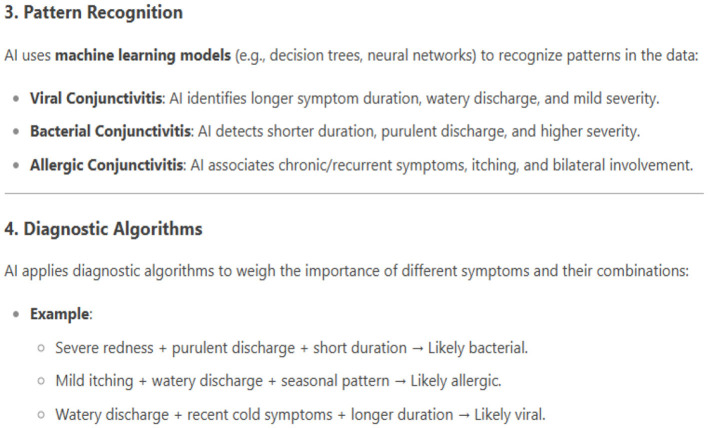
Differential diagnosis of conjunctivitis.

While AI models are showing success in healthcare, it is necessary to use them appropriately. As conjunctivitis symptoms vary from person to person, AI is helpful but cannot replace the medical professional. Sometimes, AI models trained on incomplete data may provide incorrect diagnoses. To ensure accurate diagnosis, comprehensive data on patients' medical history, lifestyle, and allergies are essential. In response to prompt 2, DeepSeek provides a roadmap for how AI tools accomplish the tasks of differentiating various types of conjunctivitis. The roadmap given by DeepSeek is very precise and accurate, including data collection using symptom duration, symptom severity, and additional features of conjunctivitis. The next phase is feature extraction, in which three prominent types of conjunctivitis, such as viral, bacterial, and allergic conjunctivitis, are used. The pattern recognition and diagnostic models follow this phase. In the following, we can see the response from DeepSeek about pattern recognition and diagnostic algorithms (Figure 3).

In addition to the abovementioned phases, DeepSeek, in its response, further explores additional data integration to improve accuracy. For example, data on patient history, environmental factors, and lab results can be used to improve the accuracy of the results. Depending on the information and processing of AI models, types of conjunctivitis are identified.

In the third prompt, we asked, “What is the best treatment for conjunctivitis infection?”. In response, we have learned about five types of conjunctivitis infections and their best treatments by ChatGPT, as given in the following Table 2.

**Table 2:** Conjunctivitis infection types and treatment.

**Sr. No**.	**Conjunctivitis infection type**	**Treatment**	**Remarks**
1.	Viral conjunctivitis	Warm compresses and artificial tears can provide relief	It takes 1–2 weeks to resolve the infection
2.	Bacterial conjunctivitis	Antibiotics, eye drops, and ointment are prescribed	A complete course of antibiotics is important
3.	Allergic conjunctivitis	Antihistamine eye drops and oral medicine can relieve the patients	No remarks
4.	Irritant conjunctivitis	Flushing the eye with clean water and	Physicians can prescribe eye drops
5.	Giant papillary conjunctivitis (GPC)	Avoid using contact lenses and eye drops	Physicians can prescribe effective medicines

Table 2 presents some key types of conjunctivitis infection along with treatment and remarks. Based on the conjunctivitis type, better treatment can be suggested to patients. However, we did not know how to spread these types, so we asked ChatGPT to reveal the causes.

In comparison with the ChatGPT, DeepSeek, in response to the prompt “What is the best treatment of conjunctivitis infection?”, revealed useful information. However, we found some common information from the two models. For instance, both DeepDeek and ChatGPT models recommend supportive care for viral conjunctivitis. However, DeepSeek recommends cold compresses for comfort, while ChatGPT favors warm compresses and reveals a resolution of 1–2 weeks. The information given by the two models for bacterial and allergic conjunctivitis aligns with each other. In summary, both models present complementary management strategies.

The fourth prompt was, “Is conjunctivitis infection dangerous and can it cause eye impairment?” The response of ChatGPT was something as follows: conjunctivitis infection is typically not dangerous but can cause discomfort and vision issues in patients. In severe cases, complications may arise, but permanent eye impairment is rare. However, practicing good hygiene and seeking prompt treatment can effectively address the issue and aid in a swift recovery. In a recent study (Jasim and Al-Jubory, 2024), the authors stated that conjunctivitis is a common type of eye condition that can cause discomfort and inconvenience. However, its effects could be minimized using appropriate prevention and management techniques.

**Figure 4:**
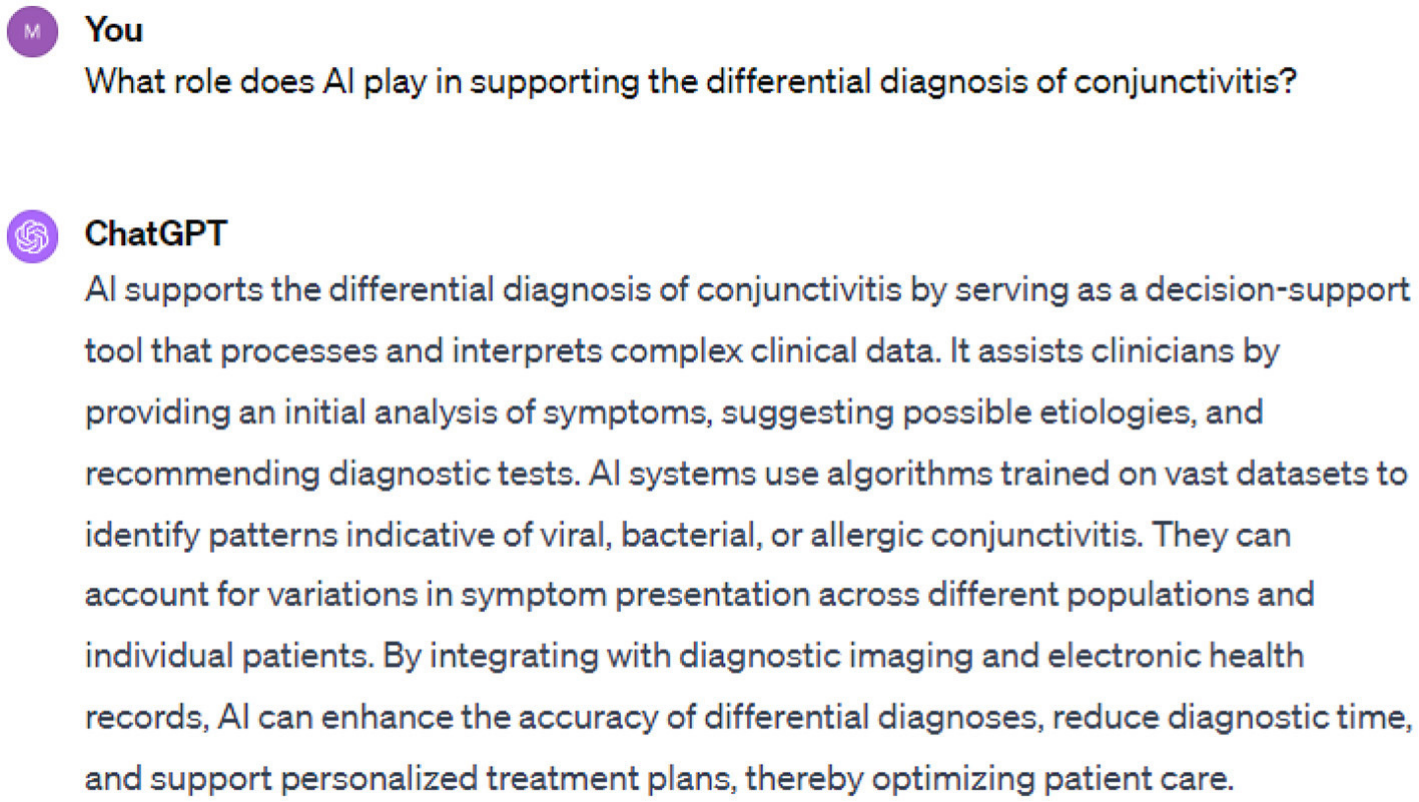
Prompt 5.

In Figure 4, we provide prompt five and its corresponding response regarding diagnostic support for the ChatGPT application.

ChatGPT and other AI tools can serve as decision-supporting tools to analyze complex clinical data. Based on the analysis and response from the ChatGPT application, some diagnostic tests can be suggested. Based on variations in symptoms of infection, AI models can identify the patterns indicating viral, bacterial, or allergic conjunctivitis. AI models are capable of accurately diagnosing the disease by integrating imaging information with electronic health records. This could result in reducing the diagnostic time and enhancing the care of patients.

**Figure 5:**
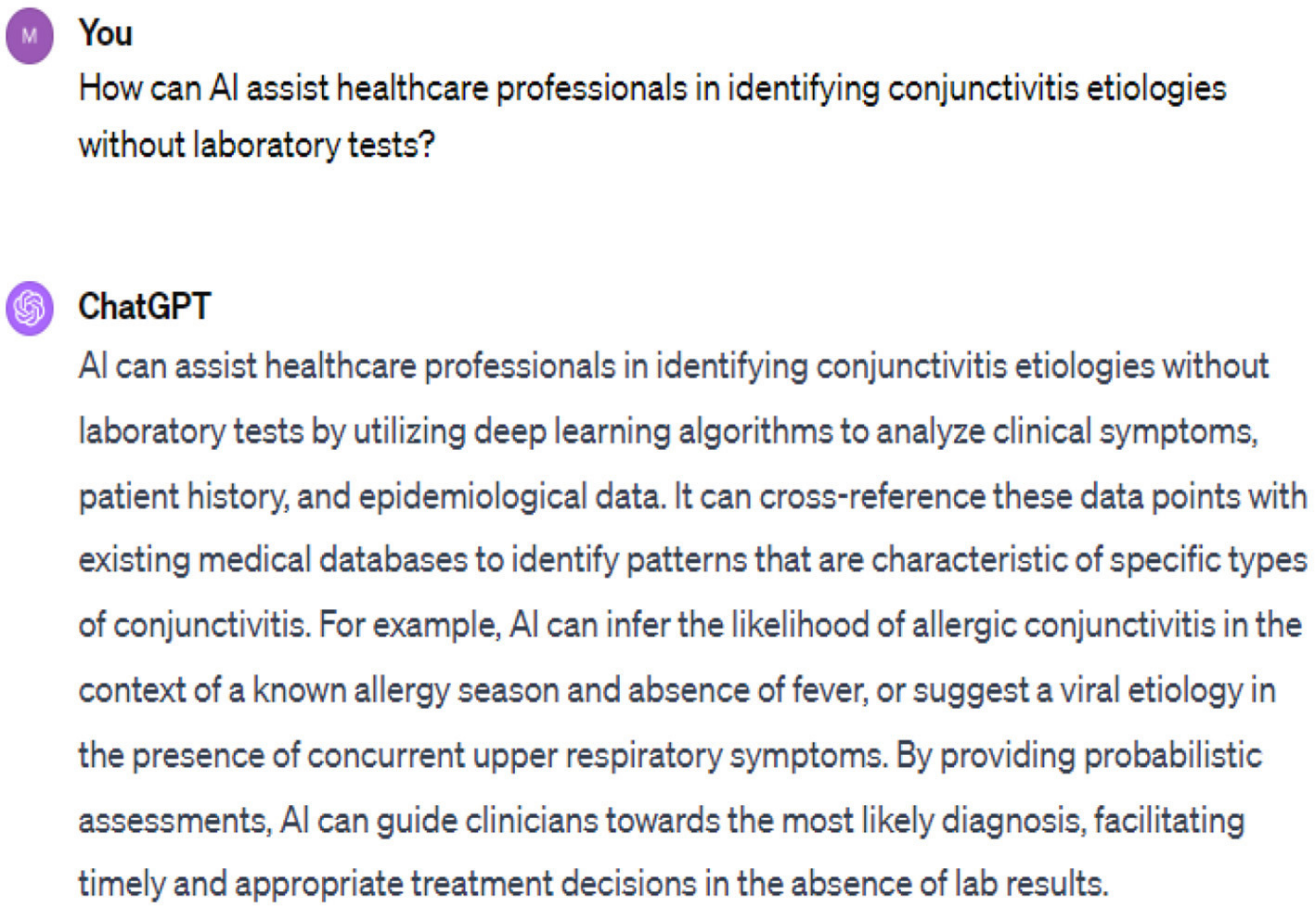
Prompt 6.

The ChatGPT application helps healthcare professionals in identifying the etiology of conjunctivitis without suggesting any laboratory test (Figure 5). For instance, an allergic type is best recognized in the context of allergy season and viral etiology in the presence of upper respiratory symptoms.

In response to a prompt “*How PubMed library is used for Conjunctivitis discussion. Cite this discussion with 30 references and provide a complete list of references*,” we received a response to this prompt, we obtained 15 and 30 references from ChatGPT and DeepSeek models, respectively. We cross-checked all references in PubMed and Google databases, from which we received the following hallucination score, as shown in Table 3.

**Table 3:** Hallucination rate of ChatGPT and DeepSeek models.

**Model**	**Total references generated**	**Fake references**	**Hallucination rate (%)**
ChatGPT	15	2	13%
DeepSeek	30	2	7%

Table 3 shows us the hallucination rate of two models in the context of references related to conjunctivitis. ChatGPT model provided us with a list of 15 references. After manually checking the list, we found some additional errors in the references. Some references did not contain a complete list of authors, while others showed incorrect issue and volume numbers. We were interested in searching for articles using their titles. Based on the titles, DeepSeek provided 30 correct references. However, we found duplicate titles in the list generated by DeepSeek.

In addition, the DeepSeek model only provided the list of titles from the PubMed database. The hallucination rate for DeepSeek was better than that of ChatGPT; notably, DeepSeek produced 30 references as were asked in the prompt. The contextual understanding of DeepSeek is superior to that of ChatGPT.

Compared to ChatGPT (13%), DeepSeek appears to be a more reliable model for generating academic references on conjunctivitis topics because of its low hallucination rate (7%). On the other hand, ChatGPT produced fewer references and a low proportion of references, which could be a concern and challenging to various stakeholders. DeepSeek is better in this performance scenario, and it becomes worrying for ChatGPT users to rely on the limited and fabricated information. Therefore, it is necessary for researchers to manually check all references to ensure academic rigor. These findings could better serve as a basis for future research or for refining prompt techniques to minimize hallucination in generating references. Prompt engineering is highly essential in highly accurate scenarios, such as medical and healthcare applications (Guo et al., 2024). However, inherent bias in training can give rise to discriminatory viewpoints, which, as a result, may produce hallucinations stemming from the overfitting of training data or lack of contextual understanding. The high rate of hallucination indicates that an AI model identifies some keywords in the prompt and aims to search for relevant references on some topics. However, it fails to find relevant and correct references. Therefore, depending only on the reference title is risky, and we need to use identifier hallucination, such as journal names or author names. The findings of our study align with a previous study, which showed a high hallucination rate for ChatGPT 3.5 and ChatGPT 4.0 in various aspects of bibliography items. Despite ChatGPT 4.0 showing an improved hallucination rate over ChatGPT 3.5, fabricated references persist.

### 4.3 Classification results

ChatGPT works on principles using contour count and mean intensity for the classification of healthy eyes and those affected by conjunctivitis in this research. The number of contour indicates the structural complexity of the eye. In conjunctivitis, the increased inflammation and vascular activity lead to a higher contour count. Mean intensity is applied to measure the overall brightness of images and closely relates with the redness of eyes. Both factors evidence the conjunctivitis.

Contour number and mean intensity factors, as shown in Table 4, help in classifying ocular images. Compared to conjunctivitis, a healthy eye exhibits fewer contours with the smooth transition. ChatGPT's classification of conjunctivitis and health images is based on the proposal of classification criteria. Contour count and mean intensity were used as factors for the classification of conjunctivitis.

**Table 4:** Classification results from ChatGPT model.

**Image**	**Contour count**	**Mean intensity**	**Classification**
10.jpg	98	113.17	Healthy
11.jpg	181	143.82	Conjunctivitis
12.jpg	430	139.59	Conjunctivitis
13.jpg	66	163.46	Conjunctivitis
15.jpg	279	152.60	Conjunctivitis
17.jpg	114	158.07	Conjunctivitis

**Figure 6:**
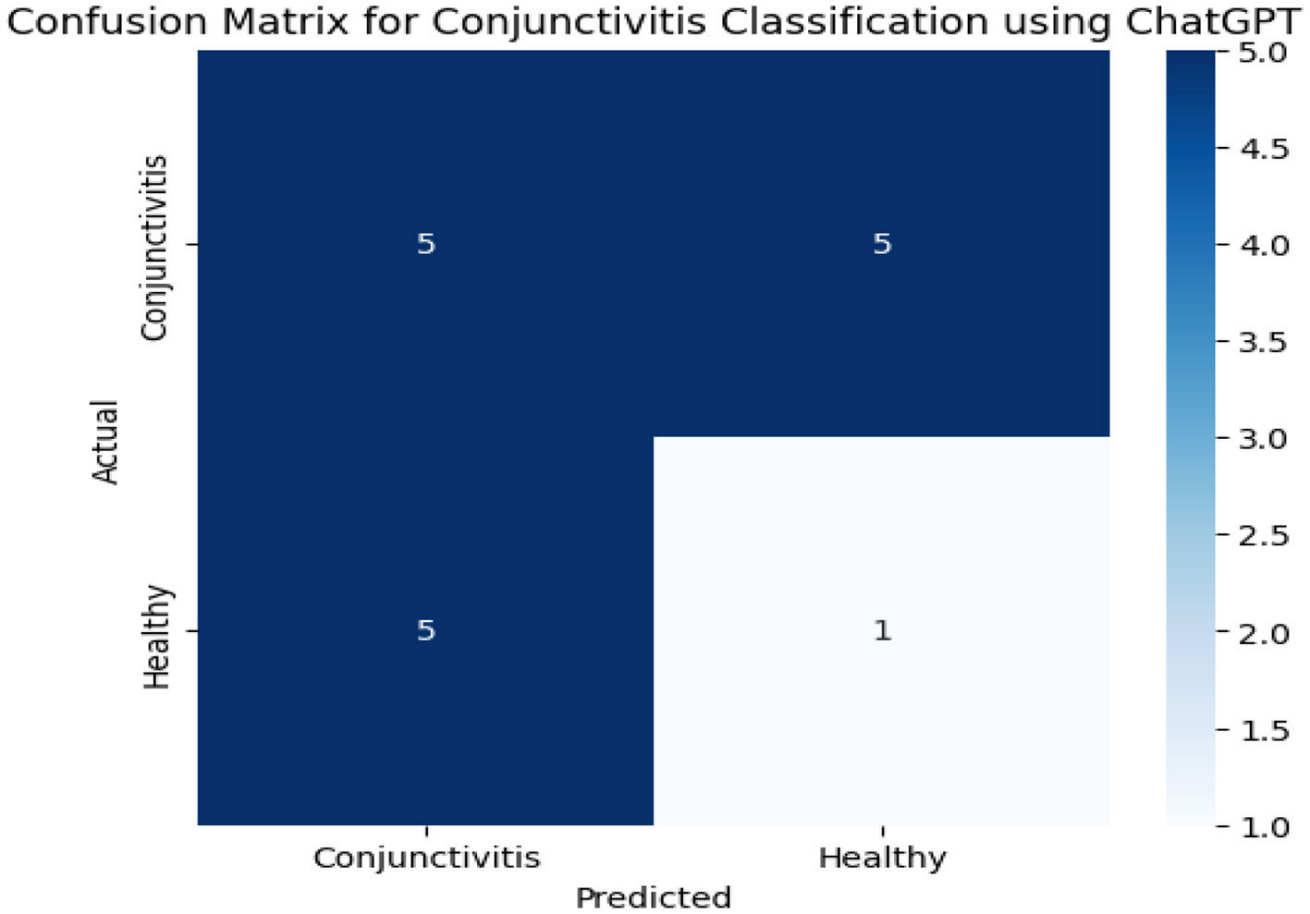
Confusion matrix results of the ChatGPT model.

Figure 6 presents confusion matrix results obtained from the ChatGPT model. We have a total of 10 instances on conjunctivitis. Out of the 10 instances, five were correctly predicted and rest of the instances were incorrectly identified. On the other hand, the ChatGPT model correctly identified only one out of six instances.

**Figure 7:**
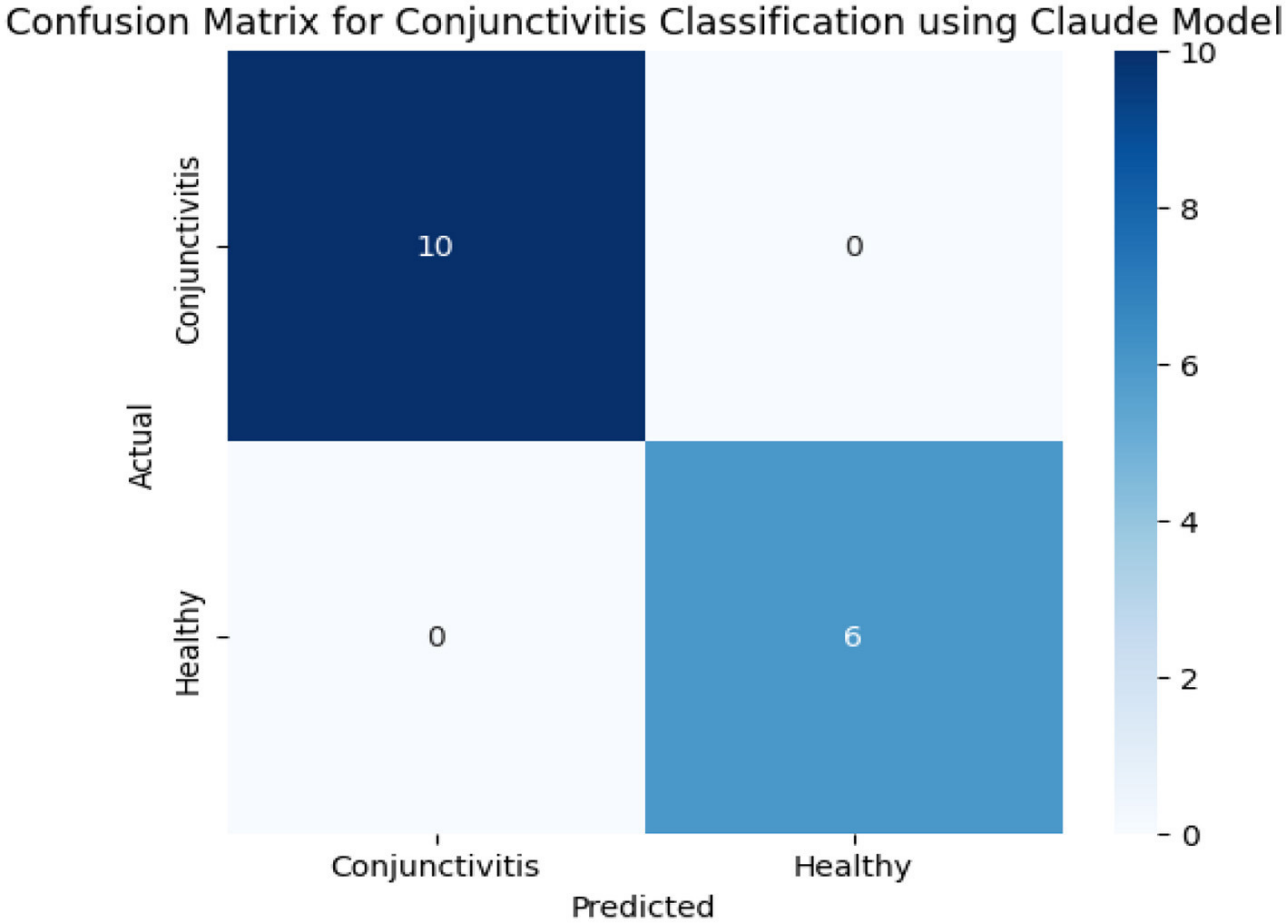
Confusion matrix results of the Claude model.

Table 5 shows us classification results of conjunctivitis from the Claude model. These results show a perfect accuracy, as images of all six healthy eyes and 10 conjunctivitis-affected eyes were correctly classified into their respective categories. Data in Table 4 and confusion matrix results in Figure 7 show no misclassification and ensure the high precision, recall, and F1-score. This reveals the effectiveness of the Claude model in distinguishing between healthy and conjunctivitis cases. Based on the contour count and mean intensity values, Claude further divided conjunctivitis cases into its sub-categories such as severe conjunctivitis, moderate conjunctivitis, and moderate–severe conjunctivitis.

**Table 5:** Claude classification results.

**Image**	**Contour count**	**Mean intensity**	**Classification**
1	5–8	95–105	Healthy
2	6–9	100–110	Healthy
3	7–10	105–115	Healthy
4	5–8	90–100	Healthy
5	8–11	100–110	Healthy
6	6–9	95–105	Healthy
7	35–40	165–175	Severe conjunctivitis
8	25–30	155–165	Moderate conjunctivitis
9	40–45	170–180	Severe conjunctivitis
10	30–35	160–170	Moderate–severe conjunctivitis
11	25–30	155–165	Moderate conjunctivitis
12	35–40	165–175	Severe conjunctivitis
13	30–35	160–170	Moderate–severe conjunctivitis
14	25–30	155–165	Moderate conjunctivitis
15	30–35	160–170	Moderate–severe conjunctivitis
16	35–40	165–175	Severe conjunctivitis

Using classification results from ChatGPT, Claude, and DeepSeek models, we obtained performance accuracy measures, as shown in Table 6.

**Table 6:** Performance comparison results.

**Model**	**Accuracy**	**Precision**	**Recall**	**F1- score**	**Specificity**
Claude	100%	100%	100%	100%	100%
ChatGPT	62.5	83.33	50.00	63.50	83.33
DeepSeek	–	–	–	–	–

The ChatGPT and Claude models show classification of dataset on conjunctivitis. DeepSeek does not favor to classify the image dataset and present some error messages when uploading images.

## 5 Discussion

We studied most recent literature on DeepSeek and other LLMs and did not find evidence on the success of DeepSeek for image classification. Several preprints of the research articles were accessed from the Google Scholar database. We even searched for articles from other databases such as ScienceDirect and Springer for related papers.

This study used only 16 images from real-world dataset containing a total of 359 images (healthy/infected eyes), but each selected case represented key conjunctivitis types, which ensured clinically relevant analysis despite the limited sample size. Each image was carefully selected by experts and represented the etiological subtypes. A small but curated dataset provided us with more meaningful performance metrics compared to a larger noisier collection. Our methodology aligns with FDA guidelines for early-stage machine learning or AI medical device validation, where small but high-quality datasets are accepted for initial capability demonstration. This is supported by a recent study that reported the sample size used for a task using LLMs can be small yet effective (Majdik et al., 2024). Furthermore, this study's results indicated that only small samples were required for substantial improvement.

In this study, we found some discrepancies in contour count and mean intensity values for yielding classification results. The inconsistencies in contour count metrics between ChatGPT and Claude arise due to differences in their underlying image processing pipelines and model-specific designs. Factors such as post-processing technique, sensitivity thresholds, and distinct edge-detection features of algorithms inherently influence contour quantification. For example, post-processing logic in noise reduction thresholds further contributes to discrepancies. ChatGPT prioritizes generalizability across various image types, while Claude's training emphasizes precision in medical imaging. However, we need to calibrate both models against controlled datasets with ground truth annotations to isolate the biases in the two models from true performance gaps.

ChatGPT and Claude models present approximate values for mean intensity while distinguishing between healthy and conjunctivitis eyes. However, two models showed strong differences in contour count values. For example, ChatGPT marked an image healthy with 98 contour count while Claude took an image as healthy with 5–11 contour count. This discrepancy may arise as each model has its own algorithm and image preprocessing phases. The Claude model might use various thresholds or edge-detection techniques to count contours and results in lower or higher counts compared to the ChatGPT's simulated metrics. In essence, unique configuration and preprocessing techniques of ChatGPT and Claude models led to different numerical outputs, and even both models were used to analyze similar features.

The most recent version of ChatGPT (ver. 4.0) has shown higher accuracy (85%) in diagnosing corneal eye disease compared to the previous version (ver. 3.5), which achieved an accuracy of 60% (Delsoz et al., 2024). Performance optimization of ChatGPT might be due to its training on more eye information records. Another study presented a comparison of ChatGPT with Isabel on the ophthalmic dataset. The role of ChatGPT as a diagnostic tool has been examined. Isabel only correctly identified one out of 10 cases, and ChatGPT identified nine cases correctly (Balas and Ing, 2023). This helps us to improve the diagnostic accuracy of health issues by learning from past cases of ophthalmic diagnosis.

A pilot study was performed to assess the capability of ChatGPT-4 for diagnosing the rare eye disease. A set of 10 treatable rare ophthalmic case studies were used in this study. The results indicated that GPT-4 could serve as a consultation tool for patients and families to obtain referral suggestions and assistance (Hu et al., 2023). However, despite these strengths, ChatGPT-4 shows limitations such as concern for patients' privacy. Moreover, ChatGPT may provide misconceptions as it was originally developed for general purposes. Later, researchers used them to make decisions in clinical diagnosis.

Claude model in conjunctivitis classification. The ChatGPT model struggles with the differentiating features, while the Claude model achieved perfect classification and correctly classified all cases with no false positives or negatives. These results are aligned with the findings of a previous study that achieved better classification accuracy for the Claude model compared to the ChatGPT model (Caruccio et al., 2024b). Our study's classification performance is overall better in comparison with the previous study. Claude 3.5 Sonnet has stronger suitability and discriminative abilities for sensitive diagnostic tasks where precision and recall are critical. However, ChatGPT requires refined prompts or supplementary context to improve reliability.

Our study is better at leveraging LLMs for analyzing conjunctivitis with etiology-specific insights, intervention, and hallucination rate quantification. The scope of a previous study is limited as it only dealt with information extraction from electronic health records about antibiotics using NLP and machine learning (ML) algorithms (Sanz et al., 2022). In a previous study, an old dataset collected between 2014 and 2018 was used. Our study employs a recent dataset on conjunctivitis that makes it more clinically relevant and representative of current trends compared to an older dataset. To evaluate ocular surface pain, an earlier study used a multimodal approach and achieved 86% accuracy from a random forest model (Kundu et al., 2022). The accuracy was inherently limited by subjective pain due to variability in imaging. In contrast to this study, the Claude model achieved 100% accuracy for conjunctivitis diagnosis. In addition, our study ensures precision in outcomes through hallucination-free LLMs.

To determine the hallucination rate, we adopted this method from a previous study (Chelli et al., 2024). Although the hallucination rate as a performance metric is widely used in the literature, its relevancy to LLMs is new and is liable for future verifications. This study employed a limited number of prompts on conjunctivitis topics, so the generalization of results might be limited and could be enhanced by proposing more prompts on conjunctivitis topics overlooked by this study. Moreover, we can expand image diversity with demographic and pathological features and incorporate a multi-centered dataset to enhance the clinical applicability of findings. Advanced data augmentation techniques could be applied to expand training set while preserving important clinical features. The current study used a set of focused prompts, which were designed to evaluate core clinical competencies. In addition, we used a rigorous and curated dataset on conjunctivitis. The proposed approach using limited prompts ensures depth over breadth, which validates real-world diagnostic reliability. Future work can expand prompts, as our methodology provides actionable and clinically relevant insights, which surpass generic evaluation. In future research, we can improve image diversity using multi-institutional collaboration on AI-based approaches to strengthen diagnostic accuracy and preserve clinical relevance.

The medical prompts used for conjunctivitis have a limited scope, which does not cover the full spectrum of conjunctivitis topics and scenarios. The proposed prompts to assess the hallucination rate of referencing by AI Chatbots may require future refinements and the introduction of AI models that are specific to the medical field. To determine the hallucination rate, we used only two databases, namely, PubMed and Google, which may have their limitations in indexing or recognizing literature, and researchers might use other search engines or databases such as Scopus and Web of Science in the future in this regard. Standardized algorithms can be used to minimize indexing biases and ensure comprehensive coverage.

## 6 Conclusion

In conclusion, this article highlights the critical situation of conjunctivitis. It discusses the role of the ChatGPT application in addressing the disease, emphasizing the importance of distinguishing between different types of conjunctivitis for accurate identification. With the ongoing advancements in AI technology, Chatbots such as ChatGPT, Claude, and DeepSeek models have the potential to mitigate the situation by delivering precise information to their users.

## Data Availability

Publicly available datasets were analyzed in this study. This data can be found at: https://www.kaggle.com/datasets/alisofiya/conjunctivitis.
